# The Relationship Between Barriers and Drivers of COVID-19 Protective Behaviors in Germany and the UK

**DOI:** 10.3389/ijph.2022.1604970

**Published:** 2022-09-08

**Authors:** Farnaz Mahdavian, George W. Warren, Darrick Evensen, Frederic E. Bouder

**Affiliations:** ^1^ Dialogik (Germany), Stuttgart, Germany; ^2^ Department of Geography, School of Global Affairs, Faculty of Social Science & Public Policy, King’s College London, London, United Kingdom; ^3^ Department of Politics and International Relations, University of Edinburgh, Edinburgh, United Kingdom; ^4^ Department of Safety, Economics and Planning, Faculty of Science and Technology, University of Stavanger, Stavanger, Norway

**Keywords:** risk perception, COVID-19, structural equation modelling, risk communication, protective behaviors

## Abstract

**Objectives:** To explore and evaluate the impact of factors including public risk perceptions on COVID-19 protective behaviors across the UK and Germany.

**Methods:** We used survey data collected from a representative sample for Germany and the UK (total N = 1,663) between April and May 2021. Using a Structural Equation Model, we evaluate the role of personal health risk perceptions, official message quality, source of news, age and political orientation on COVID-19 protective behaviors in the context of German and UK risk communication strategies.

**Results:** Personal health risk perceptions had a significant positive influence on protective behaviors. Economic risk perceptions had a negative direct influence on protective behaviors, particularly in Germany, as well as a positive indirect influence. Official message quality, use of official news sources and age had positive impacts on risk perceptions and protective behaviors. Left-wing political orientation was linked to greater likelihood of undertaking protective behaviors.

**Conclusion:** For future pandemics, more attention should be paid to evaluating and conceptualizing different varieties of risk perceptions, risk communication strategies, and demographic variables alongside their impacts on undertaking protective behaviors.

## Introduction

On 18 March 2020, Chancellor Angela Merkel emphasized that the COVID-19 pandemic was the greatest challenge Germany faced since World War II. Similar statements were made around the world as COVID-19 became an unprecedented trans-national public health crisis. As a result, a series of regulations, information and interventions have been formulated and rolled out at the national, sub-national and regional government levels, as well as by various health authority-affiliated experts [1].

National and regional governments have responded to COVID-19 virus transmission, hospitalizations and deaths with measures that have often restricted the economy and public liberties [2, 3]. At the same time attempts to minimize the risk COVID-19 have led to extensive demands for societal cooperation [4]. Authorities and different organizations around the world communicated with the public through various channels including TV, radio and newspapers. This has led to demands for the evaluation of impacts on public health or the economy [5]. During the COVID-19 pandemic, many studies have highlighted the need for improved risk communication to heighten and maintain compliance with recommended or required protective behaviors [6–8]. Simultaneously, too little has been said about the actual impact of these interventions, especially from the perspective of the complexity of various underlying patterns of perception that lead individuals to alter their behavior.

As such, this paper aims to evaluate factors that influence likelihood of undertaking relevant COVID-19 protective behaviors, comparing Germany and the UK. Using a structural equation model (SEM), we find that although personal COVID-19 health risk perception positively influences likelihood to undertake protective behaviors in both countries, economic risk perception has a statistically significant negative direct impact on protective behaviors. These findings, alongside impacts of other barriers and drivers on protective behaviors, highlight the importance of evaluating risk perceptions of viruses such as COVID-19 through more than simply the lens of personal health risk.

### Germany and the UK: Case Study

Due to the many similarities between Germany and the UK, we selected these two nations to compare government policy and societal behavior during the COVID-19 pandemic. Both nations are members of the World Health Organization and OECD. Both countries are among the world’s largest economies.

The structure of the Federal Republic of Germany is decentralized as a federal state, where 16 federal states have high levels of autonomy from their government [9]. The UK is a parliamentary democracy with three devolved governments of Northern Ireland, Scotland and Wales that have responsibility and power regarding certain policy decisions [10].

In both countries, the share of GDP on healthcare expenditure is over 10%. In Germany healthcare is mandatory for residents and is funded by statutory and private schemes. Around 88% of people hold statutory health insurance and the contribution rate is based on salary. The UK’s National Health Service (NHS) is mainly funded through taxes and free at the point of use for residents.

Before the COVID-19 pandemic, both countries had national pandemic plans for influenza which were tested during the H1N1 pandemic and revised afterward. Germany and the UK have both experienced several communicable disease outbreaks including measles and H1N1 [10].

The first confirmed COVID-19 cases in Germany and UK were announced at the end of January 2020. The first known deaths in the UK and Germany were on the 5 and 9 March respectively. The first and second waves occurred in April and November 2020 in both countries, however from mid-December 2020, the number of confirmed cases in the UK surpassed that of Germany. On 9 January 2021, the incidence rate (7-day rolling average) in the UK was 59,681, almost double as many as Germany. The daily rate of infection in the UK fell dramatically from early March until the end of May 2021, when confirmed cases were lower than seen in Germany [1, 11]. Although the cumulative trends of confirmed deaths were similar in Germany and the UK, the number of deaths in the UK were higher throughout 2020.

#### Germany

From January 2020, the Robert Koch Institute (RKI) provided information on COVID-19 statistics, situation and some of the regulations daily. The Federal Ministry of Health (Bundesministerium für Gesundheit, BMG), German States, local municipalities and other organizations also provided press conferences and almost daily updates on the guidelines, situation, regulations and German government’s actions in combating the COVID-19 pandemic [12].

In March 2020, the Federal and State Governments attempted better harmonization with joint guidelines. However, after about a month State governments were granted more freedom in self-determination at a local level [9]. Despite many similarities among states’ regulations, implementation varied between different municipalities of the same state across a wide range of NPIs.

Mass gathering restrictions began on 10 March 2020, and from 14 March different states implemented extensive nationwide closures of social spaces. These measures varied in different states, changing several times during the pandemic. Physical distancing of 1.5 m and a “contact ban” (avoiding physical meeting and contact) were announced by Federal and State governments on 22 March, and physical distancing advice continued throughout the pandemic. From 29 April 2020, mask wearing became mandatory in public areas such as shopping centers across Germany [9, 13].

#### UK

In 2020, COVID-19 communication was mostly devolved and national communication encompassing all devolved regions was only employed in occasional circumstances.

Health information about COVID-19 was provided by the NHS, while detailed statistics about the evolving situation were communicated by public health bodies. Until late June 2020, the UK Government held daily briefings. Later, the Government stated that briefings would only be held when it is required [14]. In the devolved nations daily press briefings continued for different periods of time before reducing. English COVID-19 measures changed at least 64 times between March 2020 and January 2021 [15].

Since March 2020, the government advised physical distancing of 2 m, and during 2020 people could only have close contact with the same household, extended household or bubble. On 23 March 2020 the first lockdown in the UK was announced, coming into force on 26 March 2020 where people were ordered to “stay at home.” Wearing face masks became mandatory on public transport in England on 15 June 2020, becoming mandatory in all four nations by 14 September 2020. Mitigation measures were both centralized and decentralized at different points in the pandemic, and in both Germany and the UK decisions about pandemic measures were made at the national Government level [10].

Although there were differences in the timeline and details of some mitigation measures between Germany and the UK, some of these measures were consistently employed by both authorities since the beginning of the pandemic in 2020. These include handwashing, mask wearing, avoiding physical contact, physical distancing, avoiding public spaces, gatherings, or crowds and staying at home when an individual has symptoms.

### Background

Health behavior models, such as Protection Motivation Theory [16] and the Health Belief Model [17–19], have long posited the positive impact of health risk perceptions on likelihood to undertake protective actions. In this context, health risk perceptions can be analyzed in terms of perceived susceptibility, where individuals perceive themselves as at risk of serious impact of a health hazard, or perceived high likelihood of being impacted by said hazard [17, 20].

Risk perceptions can be influenced by factors including the dread and unknown characteristics of the risk itself [21, 22]. Individual characteristics, such as socio-demographic factors, knowledge, trust, values, worldviews and political beliefs and cultural differences also influence risk perceptions [23–27].

In the context of viruses, risk perceptions are consistently found to directly impact on risk-mitigating behaviors. A meta-analysis across several pandemics underscores that severity and perceived risk of serious consequences are strongly linked to behaviors [28]. Other studies also find that public perceptions of severity and likelihood of the impact of H1N1 and H5N1 respectively were significantly related to undertaking relevant protective behaviors [29, 30].

Several studies have found relationships between increased perceived health risks of COVID-19 and greater likelihood to undertake protective behaviors across multiple countries and times during the pandemic [31–35]. Contrastingly, an Indonesian study finds that risk perceptions of COVID-19 significantly influence participants’ beliefs on protective measures, but not their actual behaviour [36]. Similarly, another study finds that health risk perception had no impact on likelihood of wearing a mask in a longitudinal survey [37].

Individuals’ economic situation and distress is also related to likelihood of greater actual exposure to risks, and increased risk severity [38–40]. Social and economic impacts of COVID-19 restrictions have resulted in negative effects on motivation [41]. In a systematic review, the practicability of any non-pharmaceutical intervention (NPI) was found to be often evaluated through the lens of economic barriers to adopting behaviors, both personally and societally [42]. A Swiss study, however, suggests that increased economic risk perceptions would negatively influence likelihood to undertake protective behaviors [35]. Economic concerns about the impact of COVID-19 should therefore be considered as potential influences on probability of undertaking protective behaviors [43].

Throughout the COVID-19 pandemic, officials have justified risk communication through an instrumental perspective [1], where communication is used to achieve specific goals and targets such as policy support or behavioral compliance [44, 45]. Communicating a clear and consistent message is recommended [46], to enhance compliance [27, 47, 48]. For instance, studies undertaken earlier in the pandemic confirm the positive impact that consistency and clarity make on COVID-19 communications [6, 49]. A Vietnamese study found a significant positive relationship between increased experience of media communication and greater engagement in protective behaviors [50]. A study of Iranian respondents found a strong positive relationship between beliefs regarding risk communication and likelihood to undertake public health behaviors [51].

Increased exposure to information sources has long been linked to heightened perceptions across many risks, and news media can amplify risks [52, 53]. Greater engagement with media reporting of the COVID-19 pandemic was linked to increased risk perceptions and protective behaviors, including vaccination intention in the UK and Australia [54, 55]. Those less likely to undertake protective behaviors against COVID-19 are also those who obtained news from official sources less frequently [56]. However, individuals are more likely to obtain COVID-19 information from news media than official sources [55].

Socio-demographic factors can also influence perceptions across a wide range of risks [23, 26]. Although factors such as age are mainly studied as control variables and tend to not be strongly influential on risk perceptions [23, 57], between-group variations in risk perceptions can occur based on the nature of the risk itself [58]. Older individuals are more vulnerable to severe consequences of COVID-19, and so one may expect heightened risk perceptions among older populations because of this. However, findings on differences between age groups’ COVID-19 risk perceptions are mixed. Although a significant relationship between older age and elevated health risk perceptions of COVID-19 has been found in Germany, this is not found among a UK sample [31]. Similarly, two additional studies do not find a relationship between age and heightened health risk perceptions in the UK and Switzerland respectively [33, 34].

Similarly mixed findings are seen in the relationship between age and economic risk perceptions. Although a study of Swiss individuals finds a positive correlation between increased age and worry about the economic situation as a result of the COVID-19 pandemic [33], a US study finds that older age groups reported lower perceived likelihood of suffering personal economic harms as a result of the pandemic [59].

Although findings on variations in COVID-19 risk perceptions between age groups have been mixed and contradictory, many studies clearly show that older individuals are more likely to undertake relevant protective behaviors, while younger people are less likely to comply and display a higher probability of undertaking more risky pandemic-related activities [54, 60–62]. These findings are consistent with past research on pandemic protective behaviors [27, 28].

Political beliefs, ideology and party affiliation are found to significantly influence perceptions and behaviors across many risks, but especially in the context of climate change beliefs [26, 63, 64]. Variation in risk perceptions and behaviors between groups with varying political beliefs is likely context- and risk-dependent [65]. Studies of German respondents found a significant relationship between political ideology and likelihood of accepting or undertaking COVID-19 protective behaviors, with those reporting as more left-wing more likely to undertake behaviors [66, 67]. Similar results are found in Italy and the UK [68, 69]. However, no relationship was found between political ideology and risk perceptions [31] and compliance with government advice [70].

Overall, the main research questions this study is trying to answer are:

(1) What factors influenced the likelihood of society undertaking COVID-19 protective behaviors (PB)?
(2) What factors influence COVID-19 health risk perception (HRP)?

### Hypotheses

Building upon the findings, and gaps in research identified above we formulated ten hypotheses that focus on three categories of H1: influential factors on COVID-19 protective behaviors, H2: COVID-19 health risk perception, and H3: COVID-19 economic risk perception.

H1.1 Health risk perception positively influences protective behaviors.

H1.2. Economic risk perception positively influences protective behaviors.

H1.3 Message quality perceptions positively influence protective behaviors.

H1.4 Public use of official news sources positively influences protective behaviors.

H1.5 Left-wing political orientation positively influences protective behaviors.

H1.6 Older age positively influences protective behaviors.

H2.1 Economic risk perception positively influences health risk perception.

H2.2 Message quality perceptions positively influence health risk perception.

H2.3 Public use of official news sources positively influences health risk perception.

H2.4 Older age positively influences health risk perception.

H3 Increasing age negatively influences personal economic risk perception.

## Methods

Data were collected via a survey distributed in Germany and the UK, in German and English respectively, between 1 April and 4 May 2021 through the online survey panel provider Qualtrics. Respondents were constrained with quotas based on census data in each nation to represent the population (over 18) based on age, gender, annual household income, and highest educational qualification. Duration of survey completion (at least 2/3 the median completion time) and correct answer to an attention filter in the survey were used as criteria for cleaning the collected data. The number of correctly completed questionnaires was 833 in Germany and 830 in the UK. As shown in Table 1, gender, age, having children at home, and immigration background of respondents were mostly similar in Germany and the UK. Differences between the two countries are seen in the number of respondents in the age range of 60–69, and the ratio of respondents in the at-risk group for COVID-19. The definition of “risk group” in this study is in line with public health authorities (see Table 1).

**TABLE 1:** Demographic distribution of respondents (Germany vs UK. 2021) (PAN-FIGHT, Germany, UK 2020–2022).

Characteristics	Germany		UK	
	Frequency	%	Frequency	%
Respondents	833		830	
Sex				
Female	421	50.5	423	51.0
Male	412	49.5	407	49.0
Age				
18–29 years	130	15.6	165	19.9
30–39 years	121	14.5	137	16.5
40–49 years	147	17.6	160	19.3
50–59 years	127	15.2	128	15.4
60–69 years	202	24.2	120	14.5
70+ years	106	12.7	120	14.5
Living with one or more people under age 17	192	23.0	207	24.9
Born outside of country of residence (respondent or one of her/his parents)	156	18.7	128	15.4
Within COVID-19 high-risk group (self-report - lung, kidney, liver or cardiovascular disease, cancer, diabetes, immunosuppressive conditions, or obesity)	393	47.2	310	37.3

**FIGURE 1:**
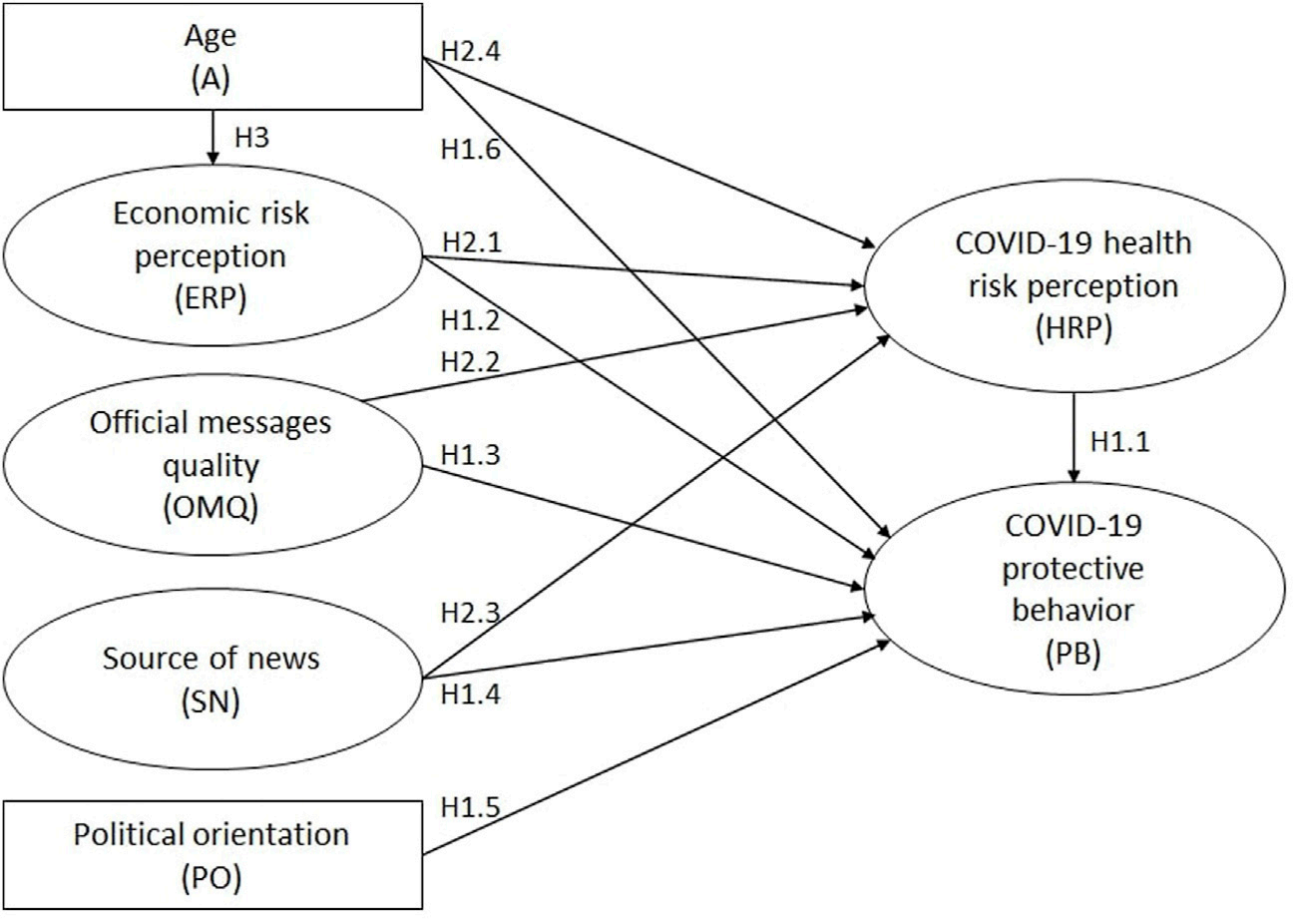
Presented model for factors influencing COVID-19 protective behaviour (Germany vs UK. 2021) (PAN-FIGHT, Germany, UK 2020–2022).

To analyze the model, a Structural Equation Model (SEM) was developed using the partial least squares (PLS) method (SmartPLS 3.3.5 program) [71]. The PLS method has the ability to handle single-item measurement, and is considered the most appropriate model for exploratory or prediction modelling [36, 72]. In the model, rectangular variables are directly measured; latent constructs (ovals) are measured by multiple indicators. Separate models were developed for each country and one Multi-Group Analysis (MGA) was undertaken to compare the countries. Figure 1 outlines the hypotheses made for this study. To see the specific question wordings and abbreviations, please refer to Table 2.

**TABLE 2:** Measurement model (Germany vs UK. 2021) (PAN-FIGHT, Germany, UK 2020–2022).

Convergent validity						Internal consistency reliability					
Constructs	Indicators	Factor loading (Bootstrap)		Average variance extracted		Cronbach’s Alpha		Composite reliability		VIF (collinearity statistics)	
		Germany	UK	Germany	UK	Germany	UK	Germany	UK	Germany	UK
COVID-19 economic risk perception			0.641	0.639	0.811	0.808	0.877	0.875			
Chance your financial situation worsens	ERP1	0.852	0.840							2,098	1,925
Chance you lose your job	ERP2	0.821	0.825							1,965	1,910
Chance your relatives or family lose their job	ERP3	0.838	0.875							1,819	2,115
Chance of major economic crisis in your country	ERP4	0.680	0.637							1,341	1,313
Source of news				0.578	0.603	0.651	0.682	0.801	0.819		
National TV networks to learn about Covid-19	SN1	0.881	0.857							1,324	1,325
National radio channels to learn about Covid-19	SN2	0.769	0.773							1,347	1,384
National or regional newspapers to learn about Covid-19	SN3	0.605	0.692							1,197	1,286
Message quality perceptions				0.855	0.872	0.830	0.853	0.922	0.932		
Clearness of information	M1	0.922	0.933							2,012	2,237
Consistency of instructions and recommendations	M2	0.926	0.934							2,012	2,237
COVID-19 health risk perception				0.816	0.847	0.887	0.910	0.930	0.943		
Chance to get corona	HRP1	0.864	0.889							2,237	2,459
Chance to be hospitalized	HRP2	0.946	0.956							4,096	5,632
Chance to die	HRP3	0.898	0.916							2,933	4,146
COVID-19 protective behavior				0.582	0.547	0.896	0.878	0.917	0.904		
Wearing mask indoors other than at home	PB1	0.635	0.516							1,492	1,254
Observing lockdown, when relevant	PB2	0.782	0.777							2,255	1,964
Keeping the required ‘social distance'	PB3	0.828	0.756							2,608	1,911
Avoiding physical contact with colleagues	PB4	0.821	0.798							2,355	2,217
Avoiding physical contact with friends	PB5	0.805	0.846							2,347	2,625
Avoiding physical contact with family in a risk group	PB6	0.726	0.796							1,880	2,196
Avoiding public spaces, gatherings, or crowds	PB7	0.807	0.781							2,177	1,978
Observing the required isolation if I have symptoms	PB8	0.675	0.582							1,618	1,382

For both the German and UK SEM models, convergent validity (see Table 2) was manifest, with most loadings close to or higher than the recommended value of 0.7 [73]. The average of variance extracted exceeded 0.5 for each construct. The thresholds of higher than 0.7 for composite reliability, and higher than 0.6 for Cronbach’s Alpha were set to ensure internal consistency reliability, which the analyses met the requirements [74].

Indicators of COVID-19 health risk perception (HRP) and economic risk perception (ERP) were collected in percentile formats developed from a study of COVID-19 risk perceptions conducted by Bruine de Bruin and Bennett [31]. Official message quality (OMQ), source of news (SN), and COVID-19 protective behaviors (PB) are measured on a 5-point Likert scale. More specifically, OMQ answers ranged from 1 (not at all clear or consistent) to 5 (extremely clear and consistent), SN from 1 (not at all using national TV, radio, and newspaper as information source for COVID-19) to 5 (several times a day) and PB from 1 (never) to 5 (always). Political orientation (PO) is scaled from very left-wing to very right-wing (scale of 1–7). Age in years ranged from 18 to 84. The protective behavior items are based on government measures to mitigate the risk over the course of pandemic outlined above. These measures were also consistently communicated to the public from official platforms. For specific question wording, see the Supplementary Details for the questionnaire.

## Results

The SEM analyses revealed similar results for Germany and the UK as expected (Supplementary Figures S1, S2). The results showed the R^2^ values (effect sizes) of ERP were 0.05 and 0.04, HRP were 0.22 and 0.28, and PB were 0.25 and 0.18 for Germany and the UK respectively. The R^2^ of HRP was higher in the UK than the Germany, however, PB in Germany was higher than the UK.

To answer the first research question, the results reveal positive significant effects (see Table 3) of HRP, OMQ, SN, PO, and age (A) on PB. In Germany, ERP has a significant effect on PB, while in the UK the effect is not significant (H1.1 – H1.6). Among factors influencing PB, ERP and PO have a negative effect. This means increasing economic risk perception negatively influences likelihood to undertake COVID-19 protective behaviors (H1.2), therefore the hypothesis is not supported, as a positive correlation was expected. People who consider themselves very right-wing are less likely to undertake COVID-19 protective behaviors (H1.5).

**TABLE 3:** Verification of proposed hypotheses - Significance level: ***p < 0.001, **p < 0.05 (Germany vs UK. 2021) (PAN-FIGHT, Germany, UK 2020–2022).

Hypothesis			Path coefficient		t-value		*p* value		f-square		Result	
			Germany	UK	Germany	UK	Germany	UK	Germany	UK	Germany	UK
Influential factors on COVID-19 protective behaviors												
H1.1	HRP	→ PB	0.204	0.142	6,690	4,209	0.000***	0.000***	0.043	0.018	Supported	
H1.2	ERP	→ PB	−0.181	−0.066	4,631	1,456	0.000***	0.145	0.034	0.004	Not Supported	
H1.3	OMQ	→ PB	0.162	0.206	4,997	5,862	0.000***	0.000***	0.033	0.047	Supported	
H1.4	SN	→ PB	0.211	0.080	5,938	2,355	0.000***	0.019**	0.050	0.007	Supported	
H1.5	PO	→ PB	-0.076	-0.128	2,019	3,869	0.044**	0.000***	0.008	0.019	Supported	
H1.6	A	→ PB	0.186	0.304	4,923	9,923	0.000***	0.000***	0.038	0.098	Supported	
COVID-19 health risk perception												
H2.1	ERP	→ HRP	0.431	0.496	12,964	14,861	0.000***	0.000***	0.225	0.317	Confirmed	
H2.2	OMQ	→ HRP	0.103	0.131	2,933	4,273	0.003**	0.000***	0.013	0.022	Confirmed	
H2.3	SN	→ HRP	0.141	0.079	4,128	2,477	0.000***	0.013**	0.022	0.008	Confirmed	
H2.4	A	→ HRP	0.140	0.099	4,488	3,529	0.000***	0.000***	0.021	0.012	Confirmed	
COVID-19 economic risk perception												
H3	A	→ ERP	−0.223	−0.186	7,625	6,185	0.000***	0.000***	0.052	0.036	Confirmed	

For the second research question, in both countries, ERP, OMQ, SN, and A have positive significant effects on HRP (H2.1 – H2.4). The last hypothesis (H3) indicates a significant negative effect of age on ERP, which indicates increasing age is negatively related to ERP.

Indirect effects of all variables on HRP and PB were significant. However, despite the negative significant direct effect of ERP on PB in Germany, ERP has an indirect positive effect on PB through HRP, although the indirect effect is > 0.1. It can be hypothesized that people with high economic risk perception might believe that COVID-19 restrictions damage the economy; therefore, their likelihood of undertaking protective behaviors is comparatively lower. However, as Öhman (2017) argues [75], if people have experienced one type of risk, that reaction can be transferred to other kinds of risk.

Except ERP on HRP, all individual variables had relatively small contributions to R^2^ values of the endogenous variables (small f^2^ value) which implies a small but significant contribution of the variables whose hypotheses were supported [74, 76].

MGA is used to explore the statistical differences between the proposed model in Germany and the UK. Despite many similarities between the results of the SEM model for Germany and the UK, Table 4 shows significant differences between these countries on three factors. There is a negative significant relationship of ERP on PB in Germany, but in UK no significant relationship exists. In Germany on average, people followed national sources of news about COVID-19 more than in the UK (2.92 and 2.49 on the scale of 1–5 respectively (Supplementary Table S1), where 1 means not at all and 5 means several times a day). Moreover, in Germany the effect of public use of SN on PB is greater than the UK (however significant in both countries). Finally, despite the significant effect of age on PB in both countries, the impact (beta coefficient) is greater in the UK.

**TABLE 4:** Partial Least Squares Multi-Group Analyses [77] – Significance level: **p < 0.05, *p < 0.1 (Germany vs. UK. 2021) (PAN-FIGHT, Germany, UK 2020–2022).

Hypothesis			Path coefficients-diff (Germany - UK)	*p* value
H1.1	HRP	→ PB	0.062	0.181
H1.2	ERP	→ PB	−0.115	0.069*
H1.3	OMQ	→ PB	−0.044	0.365
H1.4	SN	→ PB	0.131	0.016**
H1.5	PO	→ PB	0.052	0.319
H1.6	A	→ PB	−0.118	0.022**
H2.1	ERP	→ HRP	−0.065	0.174
H2.2	OMQ	→ HRP	−0.028	0.559
H2.3	SN	→ HRP	0.061	0.197
H2.4	A	→ HRP	0.041	0.342
H3	A	→ ERP	−0.036	0.379

Even after the first year of the pandemic, and despite all governments’ efforts, personal health risk perceptions were comparably low in our case study nations. However, people were generally highly likely to undertake protective behaviors (4.36 from 5-point Likert scale in both Germany and the UK). The small differences between the nations were found in PB1 and PB7, which were higher in Germany, while PB5 and PB6 were higher in the UK. In Germany people were more likely to pay greater attention to wearing masks and avoiding crowds, whereas in the UK avoiding physical contact was of the highest priority (Supplementary Table S1).

## Discussion

Both Germany and the UK had experiences with previous pandemics and their pandemic plans prior to COVID-19 were tested, however the scale of the pandemic was larger than anticipated and both faced many challenges including high fatalities and infection rates alongside mask shortages.

Average health risk perceptions differed between the two nations (23% in Germany and 19% in the UK) and were slightly lower than economic risk perceptions (26% and 27.5%). In Germany, respondents tended to follow official news sources more than UK respondents (Supplementary Table S1). In general, the content of communicated messages in the two countries were similar, but not exactly the same (Supplementary Table S1). However, public perception of the clearness and consistency of communicated messages were slightly lower among German respondents than in the UK (2.6 and 2.9 respectively out of 5). This could be due to the decentralized system of government in Germany, and points to the potential impact of different states or municipalities having different measures in place at the same time [77, 78]. Long sentences, technical terms and compound words were also found to be contributory reasons why COVID-19 related press releases by the Federal Government in Germany were difficult to understand [79].

Regarding our research questions, our model supported the significant effects of COVID-19 health risk perception, official message quality, source of news, political orientation, and age on COVID-19 protective behaviors. These results are all in line with previous findings. Importantly, the strong significant positive influence of perceptions of message quality on protective behaviors across both nations offers empirical support for theoretical assertions that COVID-19 communicators must ensure clarity and consistency to promote greater compliance [6, 49]. The finding that left-wing political orientation positively influenced likelihood of undertaking protective behaviors is consistent with findings across the UK and Germany [33, 66, 67]. This points to the need for tailored, relevant and salient message frames from trusted and respected messengers when communicating risks to politically diverse groups [80, 81].

Significant positive effects of economic risk perception, official message quality, source of news and age on COVID-19 health risk perception were also discovered. There were no significant differences between the nations (Table 4), emphasizing cross-national validity of these factors’ impact on health risk perceptions. Age positively influencing HRP in both Germany and the UK differs from similar studies [32, 34, 35], highlighting potential context-based differences related to national or temporal experiences of the pandemic. The finding that age positively influences HRP, while negatively influencing economic risk perception, emphasizes the need for measuring and evaluating a range of risk perceptions.

Economic risk perception had a significant but negative direct effect on COVID-19 protective behaviors, therefore we reject this hypothesis (H1.2). This finding varies from Siegrist et al., where no effect of economic risk perceptions on protective behaviors was found [35]. The fact that the relationship is significant in Germany, but not the UK, suggests that care should be taken when considering the impact of economic risk perceptions on COVID-19 protective behaviors across national or regional boundaries. Despite this direct negative relationship in Germany, the existing indirect positive relationship between economic risk perception and protective behaviors, mediated by COVID-19 health risk perception, could have important consequences in the wake of government communicators mainly focusing on health risks associated with COVID-19.

**FIGURE 2:**
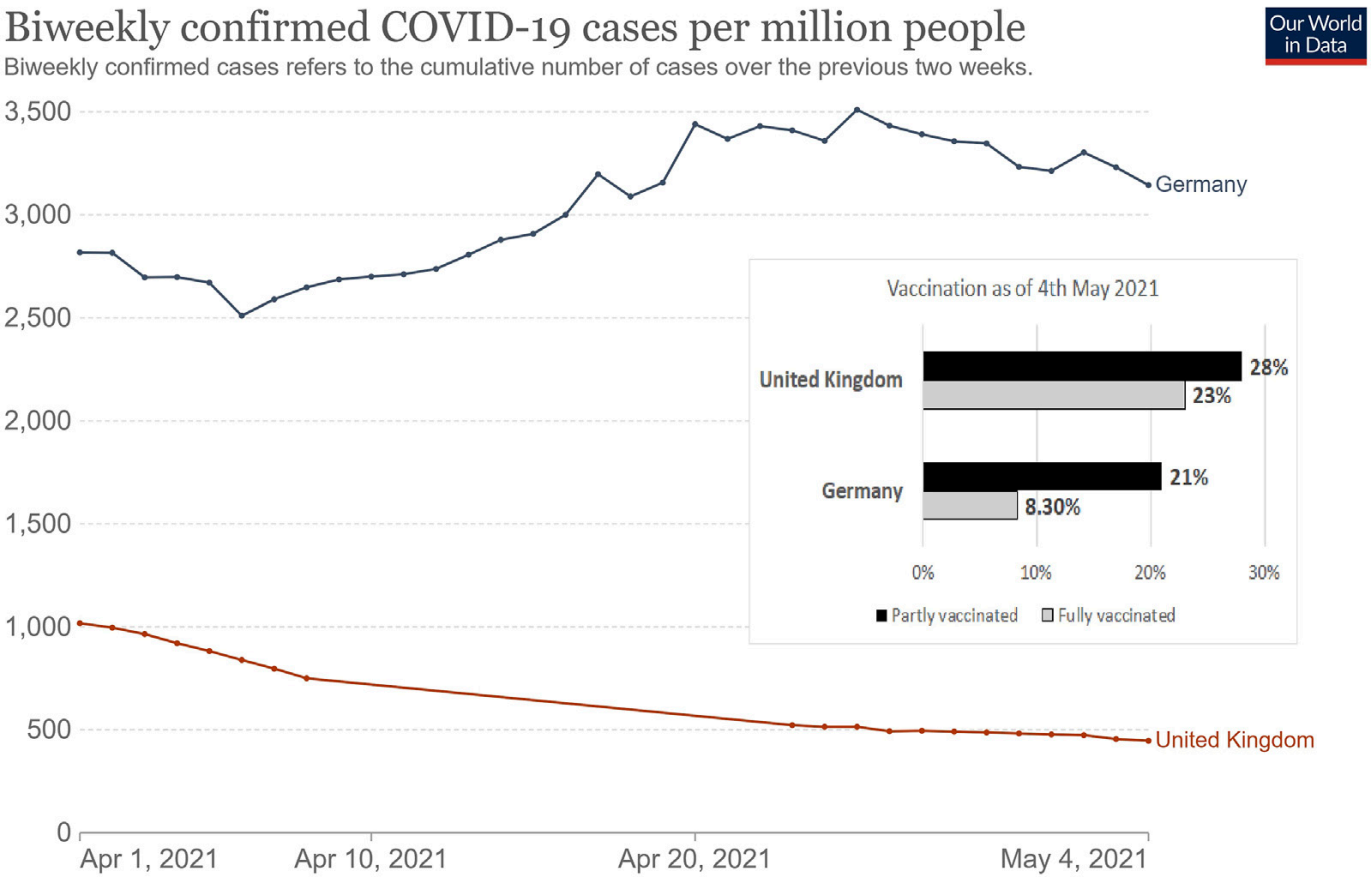
COVID-19 confirmed cases between 1st April 2021 to 4th May 2021 when our data was collected and vaccination rate by 4th May 2021 in Germany and the United Kingdom [11, 82]. Data presented by Our World in Data, licensed under the Creative Commons Attribution 4.0 International (CC BY 4.0) by Johns Hopkins University on behalf of its Center for Systems Science in Engineering (Germany vs UK. 2021) (PAN-FIGHT, Germany, UK 2020–2022).

At the beginning of the pandemic, Germany had a lower infection rate than the UK. During the first year of the pandemic, the number of confirmed cases on a 7-day rolling average were usually higher in the UK than in Germany (Supplementary Figure S3). Conversely, the UK had a higher vaccination rate than Germany while this study was conducted (51% vs. 29.5% at least partially vaccinated (Figure 2)). It is important to consider the impact of the vaccination rollout on respondents’ perceptions and optimism regarding the pandemic. According to YouGov data [83], a greater percentage of UK respondents were scared of getting COVID-19 than German counterparts in 2020 and the beginning of 2021. However, this difference narrowed after the UK started the vaccine rollout in early 2021, at a faster rate than in Germany [84]. By the end of March 2021, the rate of people fearful of catching COVID-19 was higher in Germany than in the UK. As the UK had a higher rate of vaccinated individuals than Germany when this study was conducted, this may have changed health and economic risk perceptions, views on government communication, and engagement with communicators compared to a different time in the pandemic. Similarly, German responses may have been comparatively different at a later point in time, when vaccine uptake was at a similar proportion to the UK population.

### Conclusion and Limitations

Overall, this study finds many key factors positively influencing individuals’ likelihood of undertaking COVID-19 protective behaviors, including personal health risk perceptions, official message quality, the use of official news sources, political orientation and age in both Germany and the UK. However, we discovered a negative and non-significant influence of economic risk perceptions on protective behaviors in Germany and the UK respectively. This underscores the need for a broader evaluation and consideration of risk perceptions past simply personal health risk when communicating to the public about COVID-19. This finding also cautions against applying the same risk communication strategy across multiple nations without consideration for the unique characteristics that influence protective behaviors.

Although the content of relevant COVID-19 messages communicated during the studied period were very similar in Germany and the UK [10, 12], they were not exactly the same, and this may influence public evaluations of official message quality, among other factors. Despite many similarities between Germany and the UK, there are differences in culture, religion, and political ideologies which are not taken into consideration in our evaluation of similarities and differences between the two nations.

For future pandemics, further research focus on influential factors shaping health risk perception, and their impacts on likelihood to undertake protective behaviors, is recommended. Additionally, longitudinal studies of risk perception and protective behaviors are recommended to better understand changing attitudes at different points in the pandemic. Regarding risk communication strategies, Germany could learn from how the UK government communicated to the public regarding physical distancing in the early stages of the pandemic, and the UK could learn from Germany about how to communicate to the public about mask wearing and avoiding public spaces.

The occurrence of future pandemics is inevitable. As a study of beliefs and risk perception by Attema et al. asserts, virus transmission is highly attributable to individual behaviours in the context of a pandemic such as COVID-19 [85]. To minimize virus transmission and the risk of hospitalization or death in the future, it is vital to discover not only the factors such as health risk perception which positively influenced COVID-19 protective behaviors, but also elements that could act as barriers to undertaking such protective behaviors, such as economic risk perception. Although the results of our model presented here are related to Germany and the UK, we argue that the designed model can be applied to any nation to analyze the strength of the factors in creating motivation or barriers to individual pandemic-related protective behaviors.
