# Supplementary material for: The Relationship Between Barriers and Drivers of COVID-19 Protective Behaviors in Germany and the UK
Source: Int J Public Health. 2022 Sep 8;67:1604970. doi: 10.3389/ijph.2022.1604970 (PMC9492856; doi:10.3389/ijph.2022.1604970)
Supplement: Supplementary file 1 [file DataSheet2.pdf]

Coronavirus risk communication

Start of Block: Screeners

JS

Text1 We would like to invite you to take part in this survey about risks linked to the current coronavirus (COVID-19) situation. The research is conducted by the Department of Safety, Economics and Planning at the University of Stavanger (UiS), Norway. Your participation will be very valuable to furthering understanding to improve human health. We are interested in: (1) Your perception of the risks posed by coronavirus (COVID-19) to you, to others, and to society, (2) What kinds of official guidance and instructions you received and how you responded to them, (3) Effects on your life and the people close to you, and (4) Your views on the quality of the information you received. The following text provides information on the implications of participation: 1. The survey is to be undertaken online through the Qualtrics web service. This will require approximately 15 minutes of your time. 2. You must be at least 18 years old to take part in the survey. 3. Your participation is voluntary. By completing the questionnaire, you agree to participation and give consent that the research team can use your answers for research purposes. 4. You can cancel your answers or withdraw your participation in the survey at any time by closing your browser. 5. Your answers are anonymous. This means that we will not ask for your name or other information that can identify you. We will only ask that you provide some demographic details that will help us categorize your responses and analyse them statistically. 6. The results will be treated as generalised statistical data, and they cannot be retraced back to the individual respondents. 7. Your confidentiality is safeguarded in accordance with the EU General Data Protection Regulation (GDPR) and the Norwegian Ethical Guidelines for Research. 8. Data storage will be secured with passwords and access to the data will be permitted only to certified researchers who are employed directly by the PAN-FIGHT project at UiS and associated universities. 9. If you have any comments or questions, please contact the Survey Administrator, Dr. Matan-Ilan Shapiro (matan-ilan.shapiro@uis.no). Thank you for agreeing to participate! Principal Investigators Dr. Kristin Sørung Scharffscher The University of Stavanger E-mail: Kristin.s.scharffscher@uis.no Prof. Frederic E. Bouder The University of Stavanger Email: Frederic.e.bouder@uis.no Survey AdministratorDr. Matan-Ilan ShapiroThe University of StavangerOffice: +47 51831323E-mail: matan-ilan.shapiro@uis.no

Page Break

Quota1 In what country do you live?

- Norway (1)
- Sweden (2)
- Switzerland (3)
- Germany (4)
- United Kingdom (5)
- Other (6)

Skip To: End of Block If In what country do you live? = Other

Quota2 Please provide the year of your birth.

▼ 2004 or later (104) ... 1910 (97)

Skip To: End of Block If Please provide the year of your birth. = 2004 or later

Quota3 Are you:

- Woman (1)
- Man (2)
- Trans (either man or woman) (3)
- Non-binary (4)
- Other (please specify) (5) ____

Page Break

Display This Question:

If In what country do you live? = United Kingdom

Quota4a What is your average ANNUAL household income before tax?

- Less than £13,000 (1)
- £13,000 - £18,999 (2)
- £19,000 - £25,999 (3)
- £26,000 - £31,999 (4)
- £32,000 - £47,999 (5)
- £48,000 - £63,999 (6)
- £64,000 - £95,999 (7)
- More than £96,000 (8)

Display This Question:

If In what country do you live? = Switzerland

And Q_Language = FR

Quota4b Quel est votre revenu ANNUEL moyen personnel avant impôt ?

- 0 – 26 000 CHF (1)
- 26 001 – 52 000 CHF (2)
- 52 001 – 78 000 CHF (3)
- 78 001 – 104 000 CHF (4)
- Plus de 104 000 CHF (5)

Display This Question:

If In what country do you live? = Switzerland

And Q_Language = DE

Quota4c Wie hoch ist Ihr durchschnittliches Jahreseinkommen (Brutto vor Abzug der Steuern) Persönlich?

- 0 - 26.000 CHF (1)
- 26.001 – 52.000 CHF (2)
- 52.001 – 78.000 CHF (3)
- 78.001 – 104.000 CHF (4)
- Mehr als 104.000 CHF (5)

Display This Question:

If In what country do you live? = Germany

Quota4d Wie hoch ist Ihr durchschnittliches Jahreseinkommen (Brutto vor Abzug der Steuern) Haushalt?

- Weniger als 10.800 € (1)
- 10.800 – 18.000 € (2)
- 18.001 – 24.000 € (3)
- 24.001 – 38.400 € (4)
- 38.401 – 54.000 € (5)
- 54.001 – 72.000 € (6)
- Mehr als 72.000 € (7)

Display This Question:

If In what country do you live? = Sweden

Quota4e Vad är dina inkomster PER ÅR du själv?

- Ingen egen inkomst (1)
- 1 – 149 999 SEK (2)
- 150 000 – 299 999 SEK (3)
- 300 000 – 399 999 SEK (4)
- 400 000 – 499 999 SEK (5)
- 500 000 – 699 999 SEK (6)
- Mer än 700 000 SEK (7)

Display This Question:

If In what country do you live? = Norway

Quota4f Hva er din gjennomsnittlige ÅRLIGE inntekt før skatt husstandens

- 0 – 256 000 NOK (1)
- 256 001 – 452 000 NOK (2)
- 452 001 – 666 000 NOK (3)
- 666 001 – 986 000 NOK (4)
- 986 001 – 1 526 000 NOK (5)
- Over 1 526 000 NOK (6)

Quota5 What is your highest educational qualification?

- ○ Less than secondary education completed (1)
- ○ Secondary education / high school (2)
- ○ Professional / technical diploma / apprenticeship (3)
- ○ Bachelors / undergraduate degree (4)
- ○ Masters / postgraduate degree (5)
- ○ PhD or equivalent (6)

End of Block: Screeners

Start of Block: Default Question Block

Q1 Overall, to what extent does the coronavirus disease (COVID-19) currently pose a health risk to you?

- ○ No risk at all (1)
- ○ Low level of risk (2)
- ○ Moderate level of risk (3)
- ○ Significant risk (4)
- ○ Severe risk (5)

Q1a Have you had the coronavirus disease?

- ○ No, I did not. (1)
- ○ Yes, I believe I had it, but have not specifically tested positive. (2)
- ○ Yes, I tested positive for it. (3)

Q1b Have you been vaccinated against coronavirus?

- ○ No, I have not. (1)
- ○ Yes, I received my first dose. (2)
- ○ Yes, I received two vaccine doses. (3)

Page Break

Q2 On a scale of 0 to 100 percent, what is the chance that in the next three months you will:

	Percentage chance<br>0 10 20 30 40 50 60 70 80 90 100
Get the coronavirus (1)	
Be hospitalised because of the coronavirus (2)	
Die from the coronavirus (3)	

Q3 On a scale of 0 to 100 percent, what is the chance that in the next three months:

	Percentage chance<br>0 10 20 30 40 50 60 70 80 90 100
Your financial situation will worsen (1)	
You will lose your job (2)	
Your relatives or family members will lose their jobs (3)	
There will be a major economic crisis in your country (4)	

Page Break

Q4a How would you rate the risks represented by the coronavirus (COVID-19) for your country?

	No risk at all (1)	Low level of risk (2)	Moderate level of risk (3)	Significant risk (4)	Severe risk (5)	Don't know (6)
More people falling ill than elsewhere (1)	○	○	○	○	○	○
More people dying than elsewhere (2)	○	○	○	○	○	○
Health services overstretched (3)	○	○	○	○	○	○
Deep economic crisis (4)	○	○	○	○	○	○
National debt increase (5)	○	○	○	○	○	○
Hard on small and medium size businesses (6)	○	○	○	○	○	○

Q4b How would you rate the risks represented by the coronavirus (COVID-19) for your country?

	No risk at all (1)	Low level of risk (2)	Moderate level of risk (3)	Significant risk (4)	Severe risk (5)	Don't know (6)
Loss of trust in public authorities (1)	o	o	o	o	o	o
Lack of community feeling and solidarity (2)	o	o	o	o	o	o
Attention filter: please select 'severe risk' (3)	o	o	o	o	o	o
Children missing school (4)	o	o	o	o	o	o
Other negative consequences (please specify) (5)	o	o	o	o	o	o

Skip To: End of Block If How would you rate the risks represented by the coronavirus (COVID-19) for your country? != Attention filter: please select 'severe risk' [Severe risk]

Q5 How much do the following activities put you at risk of getting coronavirus?

	No risk at all (1)	Low level of risk (2)	Moderate level of risk (3)	Significant risk (4)	Severe risk (5)	Don't know (6)
Grocery shopping (1)	○	○	○	○	○	○
Attending gatherings of more than 100 people (2)	○	○	○	○	○	○
Going to the hospital (3)	○	○	○	○	○	○
Going out for a drink or meal at a restaurant (4)	○	○	○	○	○	○
Praying in a house of worship with other community members (6)	○	○	○	○	○	○
Getting vaccinated (7)	○	○	○	○	○	○

Page Break

Q6a In the next several questions we ask you about a large number of different actions. The list is long, but is important for us to understand differences across regions and nations. Please answer as best you can.

In the UK, there are variations in how people have protected themselves against the coronavirus. How often do you use the following protection measures?

	Never (1)	Rarely (2)	Occasionally (3)	Most of the time (4)	Always (5)	Does not apply / not relevant (6
Using masks or other face cover indoors at home (1)	○	○	○	○	○	○
Using masks or other face cover indoors, other than at home (2)	○	○	○	○	○	○
Using masks or other face cover outdoors in a city or town (3)	○	○	○	○	○	○
Using masks or other face cover outdoors everywhere, including in parks and natural areas (4)	○	○	○	○	○	○
Using masks or other face cover when using public transportatio n (5)	○	○	○	○	○	○
Using industry-level face cover such as gas masks or respirator masks (6)	○	○	○	○	○	○

Q6b In the UK, there are variations in how people have protected themselves against the coronavirus. How often do you use the following protection measures?

	Never (1)	Rarely (2)	Occasionally (3)	Most of the time (4)	Always (5)	Does not apply / not relevant (6)
Washing hands with soap and water (1)	o	o	o	o	o	o
Using antibacterial liquids on hands (2)	o	o	o	o	o	o
Using antibacterial liquids on surfaces and/or routine touch points (such as door handles) (3)	o	o	o	o	o	o
Using goggles or other eye protection (4)	o	o	o	o	o	o
Observing lockdown, when relevant (5)	o	o	o	o	o	o
Opting to work from home if you can (6)	o	o	o	o	o	o
Avoiding the use of public transportation (7)	o	o	o	o	o	o

Q6c In the UK, there are variations in how people have protected themselves against the coronavirus. How often do you use the following protection measures?

	Never (1)	Rarely (2)	Occasionally (3)	Most of the time (4)	Always (5)	Does not apply / not relevant (6)
Avoiding touching your face (1)	o	o	o	o	o	o
Keeping the required 'social distance' (2)	o	o	o	o	o	o
Avoiding sharing food, dishes and drinks with others (3)	o	o	o	o	o	o
Sneezing or coughing only into your elbows (4)	o	o	o	o	o	o
Avoiding physical contact (such as shaking hands) with work colleagues (5)	o	o	o	o	o	o

Time1 Timing
First Click (1)
Last Click (2)
Page Submit (3)
Click Count (4)

Page Break

Q6d In the UK, there are variations in how people have protected themselves against the coronavirus. How often do you use the following protection measures?

	Never (1)	Rarely (2)	Occasionally (3)	Most of the time (4)	Always (5)	Does not apply / not relevant (6)
Avoiding physical contact (such as shaking hands or hugs) with close friends (1)	o	o	o	o	o	o
Avoiding physical contact (such as shaking hands or hugs) with family members (2)	o	o	o	o	o	o
Avoiding physical contact (such as shaking hands or hugs) with family members who might be in a risk group (such as the elderly) (3)	o	o	o	o	o	o
Avoiding eating at restaurants (4)	o	o	o	o	o	o

Avoiding public spaces, gatherings, or crowds (5)	○	○	○	○	○	○
Not avoiding public spaces, but observing gathering restrictions when I am with other people (6)	○	○	○	○	○	○

Q6e In the UK, there are variations in how people have protected themselves against the coronavirus. How often do you use the following protection measures?

	Never (1)	Rarely (2)	Occasionally (3)	Most of the time (4)	Always (5)	Does not apply / not relevant (6)
Creating social bubbles (consisting of members of no more than three households) (1)	○	○	○	○	○	○
Observing curfew at night (2)	○	○	○	○	○	○
Praying alone at home (as opposed to in a group setting) (3)	○	○	○	○	○	○
Staying at home when you have symptoms like sore throat (4)	○	○	○	○	○	○
Visiting elderly family members only after a negative coronavirus test (5)	○	○	○	○	○	○

Page Break

Q6f In the UK, there are variations in how people have protected themselves against the coronavirus. How often do you use the following protection measures?

	Never (1)	Rarely (2)	Occasionally (3)	Most of the time (4)	Always (5)	Does not apply / not relevant (6)
Airing rooms frequently (1)	○	○	○	○	○	○
Getting tested when having symptoms (2)	○	○	○	○	○	○
Observing the required isolation / quarantine period when I have symptoms (3)	○	○	○	○	○	○
Avoiding contact with people who could be high-risk (4)	○	○	○	○	○	○
Stockpiling food or water (5)	○	○	○	○	○	○
Cancelling a doctor's appointmen t (6)	○	○	○	○	○	○
Visiting a doctor (7)	○	○	○	○	○	○

Q6g In the UK, there are variations in how people have protected themselves against the coronavirus. How often do you use the following protection measures?

	Never (1)	Rarely (2)	Occasionally (3)	Most of the time (4)	Always (5)	Does not apply / not relevant (6)
Cancelling or postponing personal or social activities (1)	○	○	○	○	○	○
Cancelling or postponing work or school activities (2)	○	○	○	○	○	○
Cancelling or postponing air travel for pleasure (3)	○	○	○	○	○	○
Cancelling or postponing air travel for work (4)	○	○	○	○	○	○

Page Break

Q7a Again, we present a series questions about a large number of different actions. The list is long, but is important for us to understand differences across regions and nations. Please answer as best you can.
In the last seven days, have you done the following?

	Yes (1)	No (2)	Not applicable (3)
Gone out to a bar, club, or other place where people gather (1)	○	○	○
Gone to a grocery store or pharmacy (2)	○	○	○
Gone to a friend, neighbour, or relative’s residence (that is not your own) (3)	○	○	○
Had visitors such as friends, neighbours or relatives at your residence (4)	○	○	○
Attended a gathering with more than the permitted number of people, such as a reunion, wedding, funeral, birthday party, concert, or religious service (5)	○	○	○

Q7b In the last seven days, have you done the following?

	Yes (1)	No (2)	Not applicable (3)
Sought care from a hospital or health care facility (1)	○	○	○
Been placed in isolation or quarantine (2)	○	○	○
Remained in your residence at all times, except for essential activities or exercise (3)	○	○	○
Shared items like towels or utensils with other people (4)	○	○	○
Had close contact (within 2 metres) with people with whom you live (5)	○	○	○

Page Break

Q7c In the last seven days, have you done the following?

	Yes (1)	No (2)	Not applicable (3)
Had close contact (within 2 metres) with people whom do not live with you (1)	○	○	○
Gone outside to walk, hike, or exercise (2)	○	○	○
Attended a political rally, protest, or demonstration (3)	○	○	○
Attended an in-person religious service (4)	○	○	○
Travelled by airplane (5)	○	○	○
Travelled by public transportation (bus, underground, commuter rail, etc.) (6)	○	○	○

Page Break

Q8 Health authorities have recommended that people work from home as much as possible ('home office'). To what extent have you been ABLE to work from home since the outbreak of the coronavirus (COVID-19)?

- I have not been able to work from home (1)
- My ability to work from home has been fairly limited (2)
- I have been able to work from home about half of the time (3)
- I have been able to work from home most of the time (4)
- I can always work from home if necessary (5)
- I work from home anyway (6)
- Not applicable to me (7)

Q9 During the pandemic, have you ever had to work from home at the same time as home schooling or caring for children?

- Yes (1)
- No (2)
- Not applicable - I do not have children (3)

Q10 Some people have jobs that require them to interact with people face-to-face in the same location. How often does your job currently require you to come within less than one-meter distance from other people (such as customers, clients, patients, or co-workers)?

- Never (1)
- Less than once per week (2)
- Weekly (3)
- A few times each week (4)
- Daily (5)
- Several times a day (6)

Page Break

Q11a This is the third and final series of questions in which we ask for your experience of a large number of things. Thank you for your attention and patience. Please answer as best you can. In the UK, stay at home measures to curb the spread of the coronavirus are having a range of effects. To what extent have the following happened **to you** during the coronavirus pandemic, as this compares to your life before the outbreak of the pandemic?

	Much less (1)	Somewhat less (2)	Little or no change (3)	Somewhat more (4)	Much more (5)	Not applicable to me (6)
Having quality sleep (1)	○	○	○	○	○	○
Feeling free (2)	○	○	○	○	○	○
Spending time with family (3)	○	○	○	○	○	○
Having satisfying sex (4)	○	○	○	○	○	○
Time spent on hobbies (5)	○	○	○	○	○	○
Practicing indoor sports (6)	○	○	○	○	○	○

Q11b In the UK, stay at home measures to curb the spread of the coronavirus are having a range of effects. To what extent have the following happened **to you** during the coronavirus pandemic, as this compares to your life before the outbreak of the pandemic?

	Much less (1)	Somewhat less (2)	Little to no change (3)	Somewhat more (4)	Much more (5)	Not applicable to me (6)
Practicing outdoor sports (1)	○	○	○	○	○	○
Taking walks outside (2)	○	○	○	○	○	○
Attending parties or raves (3)	○	○	○	○	○	○
Spending time with my friends online (4)	○	○	○	○	○	○
Spending time with co-workers online (5)	○	○	○	○	○	○
Finding work opportunities (6)	○	○	○	○	○	○

Q11c In the UK, stay at home measures to curb the spread of the coronavirus are having a range of effects. To what extent have the following happened **to you** during the coronavirus pandemic, as this compares to your life before the outbreak of the pandemic?

	Much less (1)	Somewhat less (2)	Little to no change (3)	Somewhat more (4)	Much more (5)	Not applicable to me (6)
Receiving new side jobs (1)	○	○	○	○	○	○
Time to relax (2)	○	○	○	○	○	○
Having a healthy diet (3)	○	○	○	○	○	○
Shopping locally (4)	○	○	○	○	○	○
Concern about other global threats, such as climate change (5)	○	○	○	○	○	○

Time2 Timing
First Click (1)
Last Click (2)
Page Submit (3)
Click Count (4)

Page Break

Q11d In the UK, stay at home measures to curb the spread of the coronavirus are having a range of effects. To what extent have the following happened to you during the coronavirus pandemic, as this compares to your life before the outbreak of the pandemic?

	Much less (1)	Somewhat less (2)	Little to no change (3)	Somewhat more (4)	Much more (5)	Not applicable to me (6)
Feeling tired (1)	○	○	○	○	○	○
Mental fatigue (2)	○	○	○	○	○	○
Loneliness (3)	○	○	○	○	○	○
Anger (4)	○	○	○	○	○	○
Depression (5)	○	○	○	○	○	○

Q11e In the UK, stay at home measures to curb the spread of the coronavirus are having a range of effects. To what extent have the following happened <u>to you</u> during the coronavirus pandemic, as this compares to your life before the outbreak of the pandemic?

	Much less (1)	Somewhat less (2)	Little to no change (3)	Somewhat more (4)	Much more (5)	Not applicable to me (6)
Anxiety (1)	○	○	○	○	○	○
Sense of confusion (2)	○	○	○	○	○	○
Feeling powerless (3)	○	○	○	○	○	○
Feeling overworked (4)	○	○	○	○	○	○

Page Break

Q11f In the UK, stay at home measures to curb the spread of the coronavirus are having a range of effects. To what extent have the following happened to you during the coronavirus pandemic, as this compares to your life before the outbreak of the pandemic?

	Much less (1)	Somewhat less (2)	Little to no change (3)	Somewhat more (4)	Much more (5)	Not applicable to me (6)
Ability to separate work and home (1)	○	○	○	○	○	○
Changed the amount of money my household makes (2)	○	○	○	○	○	○
My own consumption of substances (tobacco, alcohol or drugs) (3)	○	○	○	○	○	○
My own compulsive behaviour (e.g., working out too much, cleaning, etc.) (4)	○	○	○	○	○	○
Feeling I was blamed for not adopting the right behaviour (5)	○	○	○	○	○	○
Feeling uncertain about the future (6)	○	○	○	○	○	○

Q11g In the UK, stay at home measures to curb the spread of the coronavirus are having a range of effects. To what extent have the following happened to you during the coronavirus pandemic, as this compares to your life before the outbreak of the pandemic?

	Much less (1)	Somewhat less (2)	Little to no change (3)	Somewhat more (4)	Much more (5)	Not applicable to me (6)
Worrying about the wellbeing of family members (1)	o	o	o	o	o	o
Conflicts within my household (2)	o	o	o	o	o	o
Physical aggression within my household (3)	o	o	o	o	o	o
Feeling stigmatised by others (4)	o	o	o	o	o	o
Disruption in my family situation (e.g. a breakup) (5)	o	o	o	o	o	o
Having time to engage in my hobbies or artistic activities (6)	o	o	o	o	o	o

Page Break

Q12 In the UK, some people report that during the pandemic they received help to carry essential tasks such as shopping, childcare or housework. What is the frequency with which you received essential help from the following support networks?

	Never (1)	Less than once per week (2)	Weekly (3)	A few times each week (4)	Daily (5)	Several times a day (6)	Not relevant / not applicable (7)
Relatives not living with me (1)	○	○	○	○	○	○	○
Friends not living with me (2)	○	○	○	○	○	○	○
Work colleagues (3)	○	○	○	○	○	○	○
Local volunteers and charities (4)	○	○	○	○	○	○	○
Local authorities (5)	○	○	○	○	○	○	○
Official governmental institutions such as the welfare department (6)	○	○	○	○	○	○	○
Religious organisations (such as church, mosque or synagogue support networks) (7)	○	○	○	○	○	○	○
International organisations or charity organisations (8)	○	○	○	○	○	○	○
Neighbourhood / local networks (9)	○	○	○	○	○	○	○

Page Break

Text2 We now turn to questions about the ways in which risks associated with coronavirus (COVID-19) were communicated to you.

Q13 How often have you actively sought out information related to the coronavirus (COVID-19) from official authorities?

- ○ Never (1)
- ○ Less than once per week (2)
- ○ Weekly (3)
- ○ A few times each week (4)
- ○ Daily (5)
- ○ Several times a day (6)

Q14 To what extent do you feel that the authorities in your country have met your need for information about risks associated with coronavirus (COVID-19)? (Note – communication on risks includes advice, guidance, recommendations, and official restrictions, or any other type of formal information about relevant risks.)

- ○ They have not provided any useful information whatsoever (1)
- ○ They have provided some useful information but not enough (2)
- ○ They have provided what I consider to be the correct amount of information (3)
- ○ They have provided too much information (4)
- ○ I feel overloaded with information (5)

Page Break

Q15a In the UK coronavirus-related instructions, recommendations and guidance to the public were sometimes changed or modified. Over time, were the messages that you received on the coronavirus (COVID-19) from official authorities clear and easy to understand?

- ○ Not at all clear (1)
- ○ Not very clear (2)
- ○ Somewhat clear (3)
- ○ Very clear (4)
- ○ Extremely clear (5)

Q15b Over time, do you feel that the responsible authorities have been consistent in their instructions and recommendations given to the public?

- ○ Not consistent at all (1)
- ○ Not very consistent (2)
- ○ Somewhat consistent (3)
- ○ Very consistent (4)
- ○ Extremely consistent (5)

Q16 What administrative level do you believe is BEST suited to communicate restrictions / recommendations to the public?

- ○ Local level (1)
- ○ Regional level (2)
- ○ National level (3)
- ○ Unsure / no preference (4)

Page Break

Q17 What format, in your opinion, should official authorities use to communicate their restrictions / recommendations and other information about the coronavirus?
Please select all that apply.

- ☐ Newsletter (via email or post) (1)
- ☐ Press briefing (broadcast via public communications channels, including national newspapers) (2)
- ☐ Podcast by government officials or by certified scientists (3)
- ☐ Official Government webpages (4)
- ☐ Social media posts (e.g., Facebook and Twitter) (5)
- ☐ National radio channels (6)
- ☐ National television channels (7)
- ☐ Online discussion networks (e.g., Reddit) (8)
- ☐ Other (please specify): (9) ____

Q18a How often have you used the following information sources to learn about coronavirus in the last 7 days?

	Not at all (1)	Once (2)	A few times (3)	Daily (4)	Several times a day (5)
National TV networks (1)	○	○	○	○	○
National radio channels (2)	○	○	○	○	○
National or regional newspapers (3)	○	○	○	○	○
International TV networks (from outside your country) (4)	○	○	○	○	○
International radio networks (from outside your country) (5)	○	○	○	○	○
International newspapers (from abroad) (6)	○	○	○	○	○

Page Break

Q18b How often have you used the following information sources to learn about coronavirus in the last 7 days?

	Not at all (1)	Once (2)	A few times (3)	Daily (4)	Several times a day (5)
Facebook (1)	○	○	○	○	○
Instagram (2)	○	○	○	○	○
Twitter (3)	○	○	○	○	○
Tik-Tok (4)	○	○	○	○	○
WhatsApp (5)	○	○	○	○	○
YouTube (6)	○	○	○	○	○
Podcasts (7)	○	○	○	○	○

Q18c How often have you used the following information sources to learn about coronavirus in the last 7 days?

	Not at all (1)	Once (2)	A few times (3)	Daily (4)	Several times a day (5)
Local public health officials, such as officials from your county health department (1)	○	○	○	○	○
The World Health Organization (WHO) (2)	○	○	○	○	○
Your contacts on social media (Facebook, Twitter, etc.) (3)	○	○	○	○	○
Your close friends and members of your family (4)	○	○	○	○	○
Your co-workers, classmates, or other acquaintances (5)	○	○	○	○	○
Your physician, doctor, or pharmacist (6)	○	○	○	○	○
Other medical scientists (7)	○	○	○	○	○

Page Break

Q18d How often have you used the following information sources to learn about coronavirus in the last 7 days?

	Not at all (1)	Once (2)	A few times (3)	Daily (4)	Several times a day (5)
A religious leader or faith group peers (1)	○	○	○	○	○
Your workplace management (2)	○	○	○	○	○
Public opinion leaders and influencers (3)	○	○	○	○	○
Health charities and other civil society organisations (4)	○	○	○	○	○
Official guidance provided by authorities of other countries, through their media channels (internet, TV, radio, etc.) (5)	○	○	○	○	○

Q18e How often have you used the following information sources to learn about coronavirus in the last 7 days?

	Not at all (1)	Once (2)	A few times (3)	Daily (4)	Several times a day (5)
People on social networks whom I do not know (1)	o	o	o	o	o
Non-medical scientists (2)	o	o	o	o	o
Neighbourhood and/or apartment block committees (3)	o	o	o	o	o
Alternative sources who tend to go against official advice (4)	o	o	o	o	o

Page Break

Q19a In the UK, some people have expressed concerns that some family members may not have access to important official information about the coronavirus (COVID-19).

If you have family members in the following categories, how accessible do you believe is the official information to them?

	Totally inaccessible (1)	Quite inaccessible (2)	Somewhat accessible (3)	Reasonably accessible (4)	Fully accessible (5)	Don't know (6)	Not applicable (7)
Children aged 0-10 years (1)	○	○	○	○	○	○	○
Children aged 11-18 years (2)	○	○	○	○	○	○	○
Elderly, aged 65-85 years (3)	○	○	○	○	○	○	○
Elderly, aged 85 years and older (4)	○	○	○	○	○	○	○
Male family members irrespective of age (5)	○	○	○	○	○	○	○
Female family members irrespective of age (6)	○	○	○	○	○	○	○

Q19b In the UK, some people have expressed concerns that some family members may not have access to important official information about the coronavirus (COVID-19).

If you have family members in the following categories, how accessible do you believe is the official information to them?

	Totally inaccessible (1)	Quite inaccessible (2)	Somewhat accessible (3)	Reasonably accessible (4)	Fully accessible (5)	Don't know (6)	Not applicable (7)
People with hearing impairments (1)	○	○	○	○	○	○	○
People with visual impairments (2)	○	○	○	○	○	○	○
People with other disabilities (3)	○	○	○	○	○	○	○
Adult family members with limited language proficiency (4)	○	○	○	○	○	○	○

Page Break

Text3 The survey will now finish with several questions that help us compare responses across different sub-populations in your country, and across countries.

Page Break

Q20 Were you born in the country where you live now?

○ Yes (1)

○ No (2)

Q21 Was your mother born in this country?

○ Yes (1)

○ No (2)

○ Don't know / prefer not to answer (3)

Q22 Was your father born in this country?

○ Yes (1)

○ No (2)

○ Don't know / prefer not to answer (3)

Display This Question:

If Were you born in the country where you live now? = No

Q23 In which country were you born?

____

Page Break

Q24 What is your relationship status?

- ◯ Single (1)
- ◯ Married / civil partnership (2)
- ◯ Cohabitation (3)
- ◯ Divorced (4)
- ◯ Widowed (5)
- ◯ Other (please specify) (6) ____

Q25 Please select the category below that best fits your current household situation.

- ◯ Renting alone (1)
- ◯ Renting in a flat share (2)
- ◯ Renting with my family (3)
- ◯ Single owner of an apartment / house (4)
- ◯ Shared ownership of apartment / house (with family / spouse / relatives) (5)
- ◯ Other (please specify) (6) ____

Q26 Please indicate how many persons in the following age groups, including you, live in your home:

	0 (1)	1 (2)	2 (3)	3 (4)	4 (5)	5 (6)	More than 5 (7)
0-10 years (1)	○	○	○	○	○	○	○
11-18 years (2)	○	○	○	○	○	○	○
19-30 years (3)	○	○	○	○	○	○	○
31-40 years (4)	○	○	○	○	○	○	○
41-50 years (5)	○	○	○	○	○	○	○
51-60 years (6)	○	○	○	○	○	○	○
61-70 years (7)	○	○	○	○	○	○	○
71-80 years (8)	○	○	○	○	○	○	○
81+ years (9)	○	○	○	○	○	○	○

Page Break

Q27 What is the approximate size of the town / city / village where you live?

- ○ Fewer than 5,000 people (1)
- ○ 5,000 - 9,999 (2)
- ○ 10,000 - 49,999 (3)
- ○ 50,000 - 99,999 (4)
- ○ 100,000 - 499,999 (5)
- ○ 500,000 - 999,999 (6)
- ○ 1,000,000 - 2,999,999 (7)
- ○ 3,000,000 or more (8)
- ○ I don't know (9)

Q28 Which of the following best describes your current job status?

- ○ Employed Full-Time (1)
- ○ Employed Part-Time (2)
- ○ Self-employed or freelance (3)
- ○ Retired / pensioner (4)
- ○ Unemployed (looking for work) (5)
- ○ Unemployed (not looking for work) (6)
- ○ Unpaid housework with no outside employment (7)
- ○ Student (8)
- ○ Other unpaid work (e.g., volunteer) (9)
- ○ Other (please specify) (10) ____

Page Break

Q29a According to public health authorities, people with certain conditions are likely to show a severe course of illness from Covid-19. Do any of the following conditions apply to you, or to any member of your household?

	No (1)	Yes (2)
chronic lung disease (like COPD), lung cancer, or lung transplantation (1)	○	○
cardiovascular system disease (like coronary hearth disease or high blood pressure) (2)	○	○
diabetes (3)	○	○
liver disease (4)	○	○

Q29b According to public health authorities, people with certain conditions are likely to show a severe course of illness from Covid-19. Do any of the following conditions apply to you, or to any member of your household?

	No (1)	Yes (2)
kidney disease (1)	○	○
cancer (2)	○	○
Immunosuppressive conditions or treatments (e.g., cortisone) (3)	○	○
obesity / adipositas (4)	○	○
smoking (5)	○	○

Page Break

Q30 Do you consider yourself a member of any religious organisation, spiritual community, or faith group?

○ No (1)

○ Yes (2)

Display This Question:

If Do you consider yourself a member of any religious organisation, spiritual community, or faith gr... = Yes

Q31 Please indicate the name of this religious or spiritual group.

____

Q32 If you have a religion, what is the level of your involvement in worship practice?

○ Daily (1)

○ Weekly (2)

○ Monthly (3)

○ Once every few months (4)

○ Very seldom (5)

○ Not applicable (6)

Page Break

Q33 Sometimes people describe themselves as more right-wing or more left-wing. Please let us know where you would place yourself on this spectrum.

- very left-wing (1)
- moderately left-wing (2)
- left-leaning (3)
- centrist (4)
- right-leaning (5)
- moderately right-wing (6)
- very right-wing (7)

Page Break

Final If you have any final comments, please write them below, or contact Dr Matan-Ilan Shapiro (Department of Safety, Economy and Planning, University of Stavanger) at matan-ilan.shapiro@uis.no.
You can comment on the survey or add any additional information you think might be important for our analysis of your answers.
Finally, in the coming months we will be conducting interviews with members of the public to learn more on people's opinions concerning Covid-19 risk communication. If you would like to participate in such follow-up interview, please send an email to the Survey Administrator Dr. Matan Shapiro (matan-ilan.shapiro@uis.no). We will get back to you and schedule an online interview, which will be held in your native language. We will protect your identity and will never publish any identifying detail on you.
To have your responses recorded and complete this survey, please click the last 'next' button.

____

____

____

____

____

Page Break

End of Block: Default Question Block
