# Supplementary material for: The Relationship Between Barriers and Drivers of COVID-19 Protective Behaviors in Germany and the UK
Source: Int J Public Health. 2022 Sep 8;67:1604970. doi: 10.3389/ijph.2022.1604970 (PMC9492856; doi:10.3389/ijph.2022.1604970)
Supplement: Supplementary file 2 [file DataSheet1.docx]

**Supplementary materials**


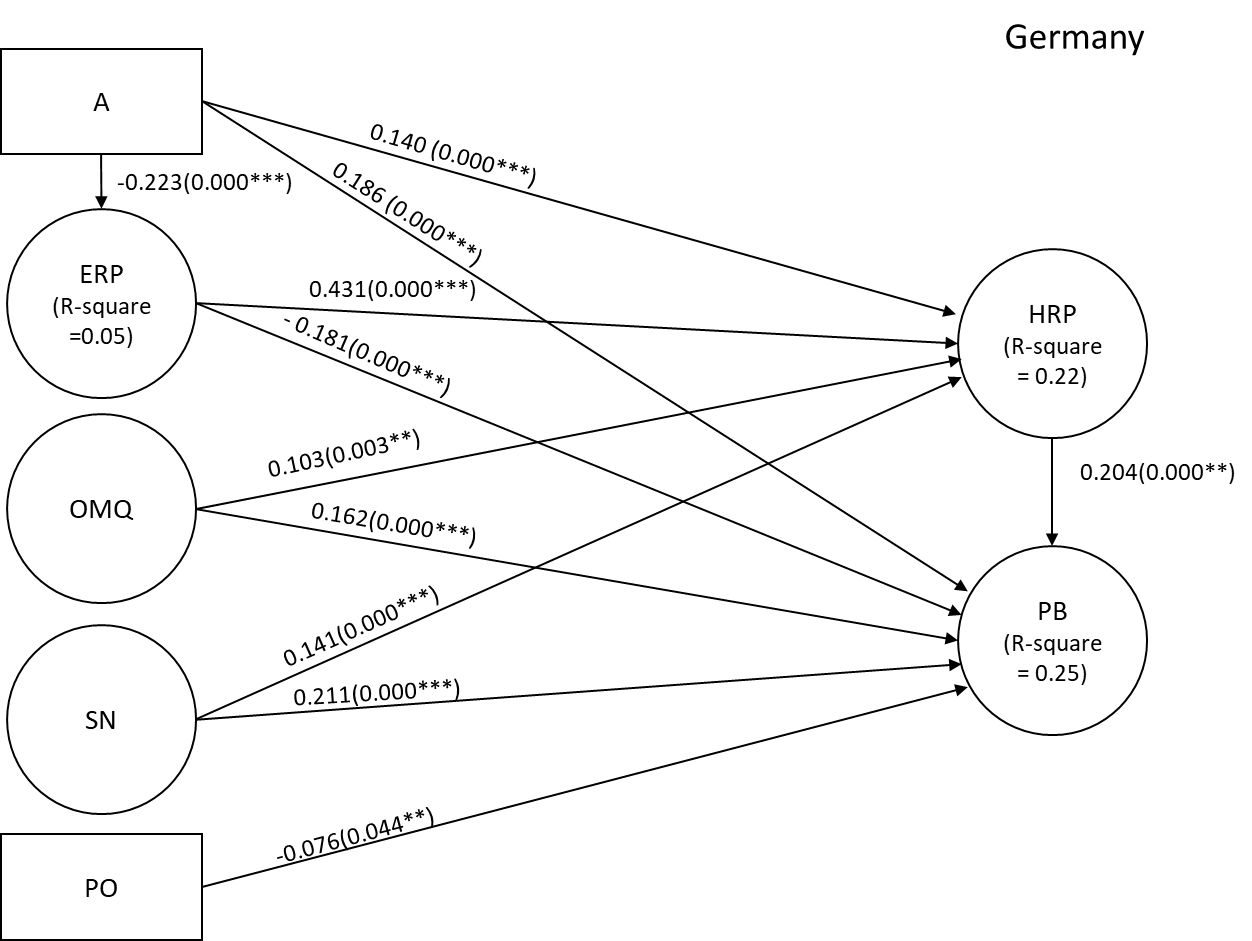


Figure S1 Germany - Structural Equation Modeling results of path coefficient and p-value (Significant level: ***p < 0.001, **p < 0.05)


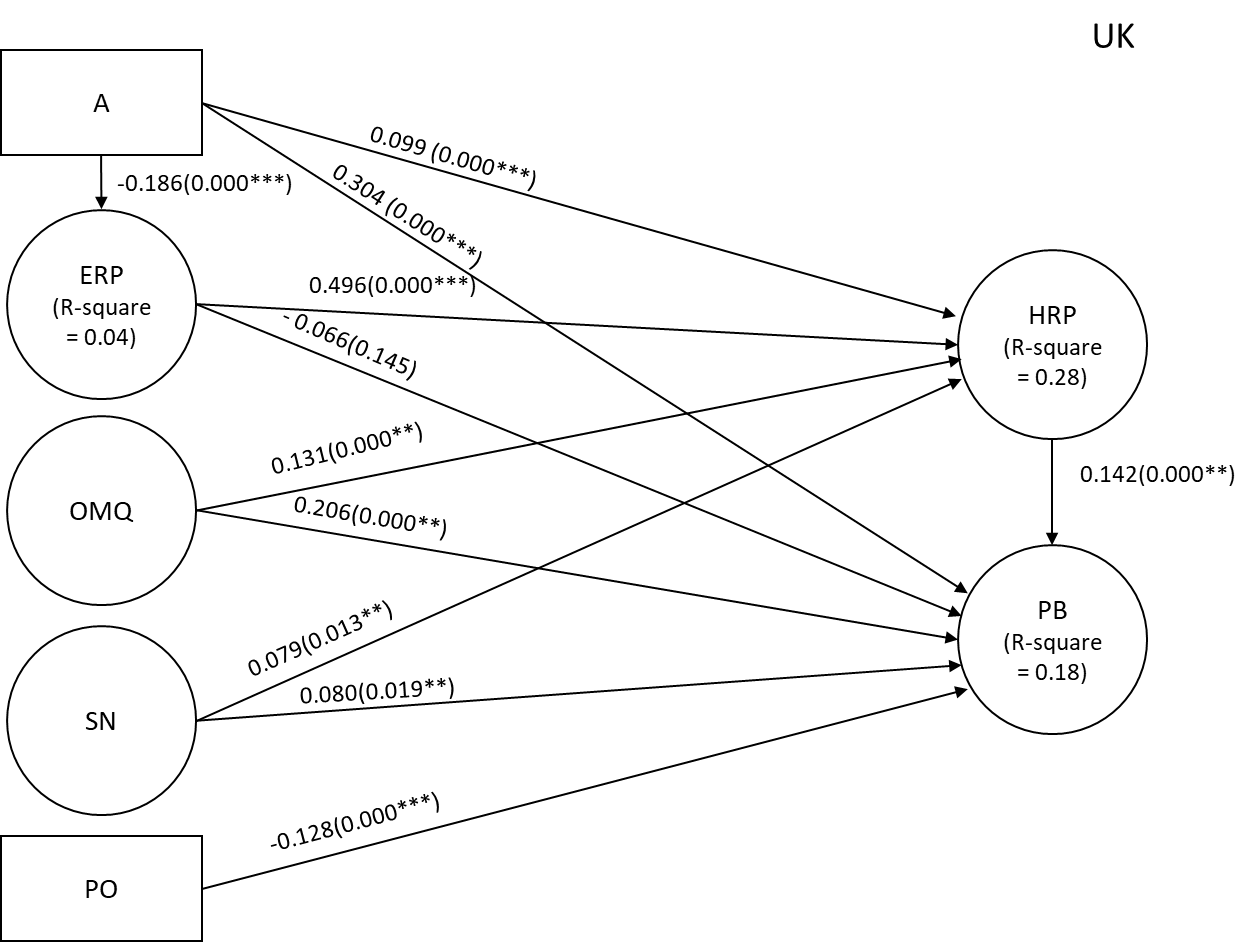


Figure S2 UK - Structural Equation Modeling results of path coefficient and p-value (Significant level: ***p < 0.001, **p < 0.05)


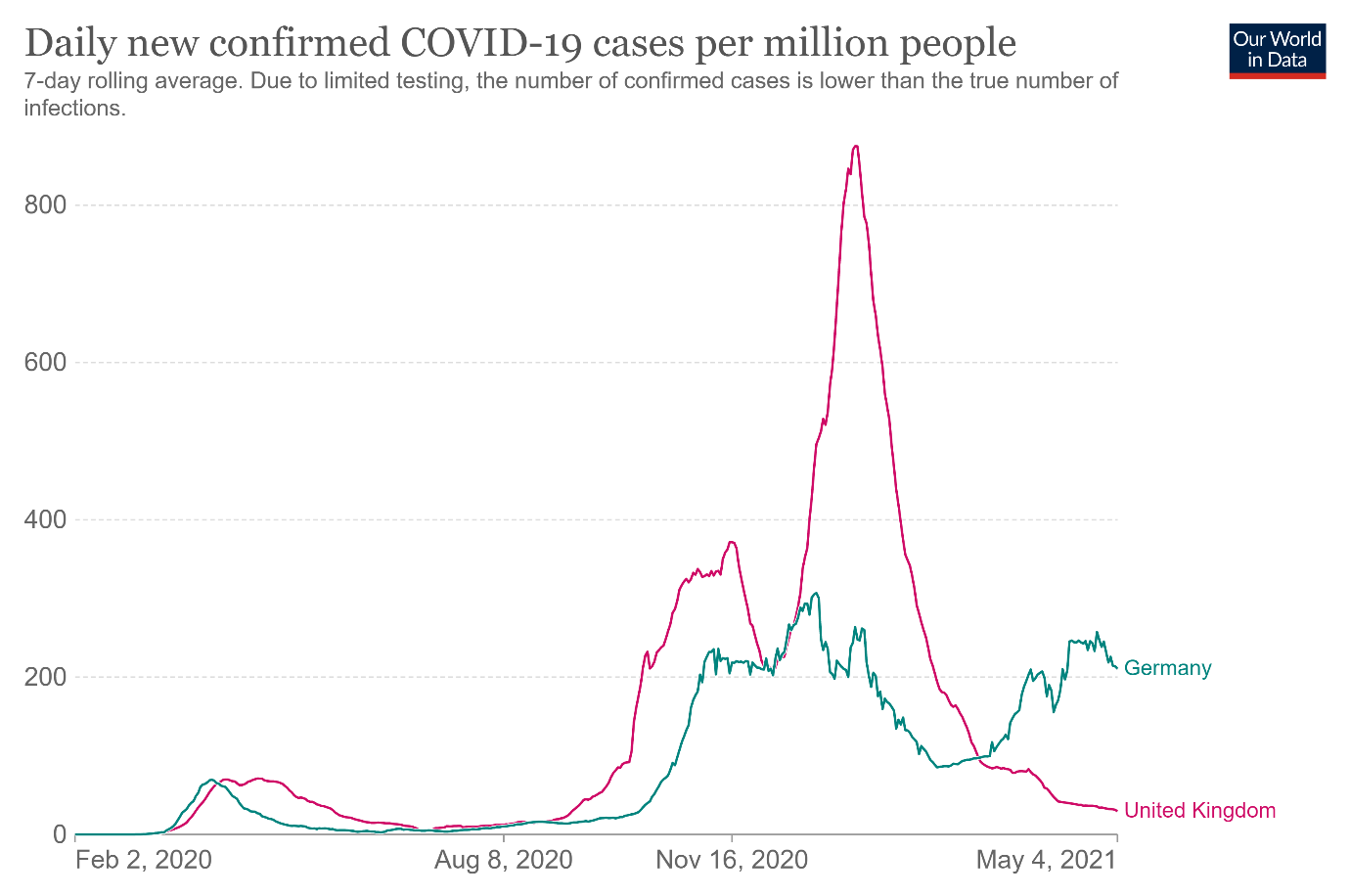


Figure S3 Daily confirmed Covid-19 cases in Germany and the United Kingdom from January 2020 till 4th May 2021 when our data were collected [66]

| Table S1 Mean analysis of indicators |  | **Responses mean** | |
| --- | --- | --- | --- |
|  | **Indicators** | **German** | **UK** |
| ***Covid-19 economic risk perception*** | **ERP** | **26.0%** | **27.5%** |
| *Chance your financial situation worsen* | ERP1 | 27.6% | 27.8% |
| *Chance you lose your job* | ERP2 | 13.7% | 15.9% |
| *Chance your relatives or family lose their job* | ERP3 | 21.0% | 22.5% |
| *Chance of major economic crisis in your country* | ERP4 | 41.9% | 43.9% |
|  |  |  |  |
| ***Source of news^[[1]](#footnote-1)^ on COVID-19 (frequency of use in the last 7 days)*** | **SN** | **2.92** | **2.49** |
| *National TV networks to learn about Covid-19* | SN1 | 3.32 | 3.05 |
| *National radio channels to learn about Covid-19* | SN2 | 2.85 | 2.18 |
| *National or regional newspapers to learn about Covid-19* | SN3 | 2.6 | 2.23 |
|  |  |  |  |
| ***Message quality perceptions*** | **OMQ** | **2.60** | **2.90** |
| *Clearness of information* | OMQ1 | 2.69 | 3.06 |
| *Consistency of instructions and recommendations* | OMQ2 | 2.48 | 2.72 |
|  |  |  |  |
|  |  |  |  |
| ***Covid-19 health risk perception*** | **HRP** | **23.1%** | **19.3%** |
| *Chance to get corona* | HRP1 | 30.1% | 25.9% |
| *Chance to be hospitalized* | HRP2 | 22.3% | 18.0% |
| *Chance to die* | HRP3 | 17.0% | 13.8% |
|  |  |  |  |
| ***Covid-19 protective behaviour*** | **PB** | **4.36** | **4.36** |
| *Wearing mask indoors other than at home* | PB1 | 4.34 | 4.06 |
| *Observing lockdown, when relevant* | PB2 | 4.63 | 4.52 |
| *Keeping the required 'social distance'* | PB3 | 4.3 | 4.38 |
| *Avoiding physical contact with colleagues* | PB4 | 4.48 | 4.52 |
| *Avoiding physical contact with friends* | PB5 | 4.16 | 4.35 |
| *Avoiding physical contact with family in a risk group* | PB6 | 4.12 | 4.40 |
| *Avoiding public spaces, gatherings, or crowds* | PB7 | 4.48 | 4.27 |
| *Observing the required isolation if I have symptoms* | PB8 | 4.35 | 4.36 |

Table S2 Key communicated messages in Germany and the United Kingdom in 2020

| **Germany** | | | |
| --- | --- | --- | --- |
| **Message** | | **Translation** | **Details** |
| Die AHA+A+L-Formel: Abstand wahren, auf Hygiene achten und – da, wo es im Alltag eng wird – eine Maske tragen. Zusätzlich sollten wir im Alltag die Corona-Warn-App nutzen und in Innenräumen regelmäßig lüften. | | the AHA + A + L formula: keep your distance, pay attention to hygiene and - where things get tight in everyday life - wear a mask. In addition, we should use the Corona warning app in everyday life and ventilate the rooms regularly. | The original acronym “AHA” stands for Abstand, Hygiene, Alltagsmaske. The acronym was extended to “AHA + A + L” with A standing for the COVID-19 tracing app and L standing for air (Lüften) . |
| “Zusammen gegen Corona” | | “Together against Corona” |  |
| “Wir bleiben zuhause!” | | “We’re staying home” |  |
| “Wir halten zusammen” | | “We stay together” |  |
| “Applaus für die Helden” | | “Applause For The Heroes” |  |
| "Vermeiden Sie öffentliche Verkehrsmittel und unnötige Reisen" | | “Avoid public transport and unnecessary travel” |  |
| "Reduzieren Sie persönliche Kontakte" | | “Reduce personal contacts” |  |
| "Home-Office-Möglichkeiten nutzen" | | “Use home office options” | Encouragement to work from home if it is possible |
| **UK** | | | |
| **Message** | **Details** | | |
| “Stay home. Protect the NHS. Save Lives” | Stay at home guidance to population to protect the National Health Service (NHS) and to save lives.  Used for different periods in each of the nations. Message first used from the end of March 2020 (GOV.UK, 2020a). Used in England during the first wave of the pandemic but England dropped this message in May 2020 when easing lockdown restrictions. | | |
| “Stay alert, control the virus, save lives” | Used from May 2020 in wave 1 in England only, replacing the stay home messaging. The other nations did not change from the general stay at home guidance (BBC, 2020b). | | |
| Hands. Face. Space. | Ran from September 2020 onwards (GOV.UK*, 2020e). This message was shown on the podiums during press briefings and featured in COVID-19 communication materials. Key mitigation actions: wash hands, cover face and maintain space between you and persons outside of your household. | | |
| “Rule of 6” | Used in England when the rule of 6 was implemented in September 2020 for meeting others with whom you do not live (GOV.UK, 2020b) | | |
| “We all must do it to get through it” | Used in Northern Ireland from March until end of 2020 (The Executive Office*, 2020). Slogan used throughout 2020. The slogan was also accompanied pictographs of either some of the key mitigation actions “stay home”, “keep distance”, “wash hands” or additional phrases “stay safe” “save lives”. | | |
| “FACTS”  “Living with FACTS helps keep us safe.”  “Remember FACTS”  “Remember FACTS for a safer Scotland” | Used in Scotland. FACTS campaign was launched in June 2020 and used until end of 2020 (Gov.scot, 2020c). The campaign was to raise awareness of the key actions members of the public should continue to take.  The acronym “FACTS” stands for:  F – Face covering  A – Avoid crowds  C – Cleaning hands  T – Two meter distance  S – Self-isolate  The acronym summarises the key things people should do to help minimise the spread. | | |
| “Stay safe. Protect others. Save lives” | Used in Scotland. First seen in a press conference in June 2020, aligning with the launch of the FACTS campaign and moving from the stay at home message (Gov.scot, 2020b). | | |
| “Stay home. Stop the spread. Save lives.” | Used in Scotland. A variant on the stay home messaging. | | |
| “Stopping the spread starts with all of us.” | Used in Scotland. Shown at press briefings from 2^nd^ November 2020, coinciding with the launch of the Covid Protection Levels (Gov.scot, 2020a). | | |
| “Stick with it Scotland, for yourselves and each other”  #WeAreScotland | Used in Scotland from July 2020. | | |
| “NHS is open” | Used in Scotland to encourage the use of the NHS during the pandemic and not to put off treatment due to the pandemic. | | |
| “Keep Wales safe”  “Together we’ll keep Wales safe” | Used in Wales. | | |
| “Three rules to keep Wales safe” | Used in Wales. Washing hands, wearing a face covering and keeping 2m apart. | | |

1. Answers: 1=Not at all, 2=Once, 3=a few times, 4=Daily, 5=Several times a day [↑](#footnote-ref-1)
